# Amputation of Cervix Uteri

**Published:** 1878-06

**Authors:** 


AMPUTATION OF CERVIX UTERI.—By W. H. Wathen, M.D., Clinical Lecturer on Diseases of Women and Children, Louisville Medical College, etc. Read before the Kentucky State Medical Society, April 3, 1878. (Reprint from May No. *Richmond and Louisville Medical Journal.*)

